# C3orf70 Is Involved in Neural and Neurobehavioral Development

**DOI:** 10.3390/ph12040156

**Published:** 2019-10-16

**Authors:** Yoshifumi Ashikawa, Takashi Shiromizu, Koki Miura, Yuka Adachi, Takaaki Matsui, Yasumasa Bessho, Toshio Tanaka, Yuhei Nishimura

**Affiliations:** 1 Department of Integrative Pharmacology, Mie University Graduate School of Medicine, Tsu, Mie 514-8507, Japan; thegracioso1113@gmail.com (Y.A.); tshiromizu@doc.medic.mie-u.ac.jp (T.S.); 318090@m.mie-u.ac.jp (K.M.); 319m001@m.mie-u.ac.jp (Y.A.); 2 Gene Regulation Research, Graduate School of Biological Sciences, Nara Institute of Science and Technology, Takayama, Nara 630-0192, Japan; matsui@bs.naist.jp (T.M.); ybessho@bs.naist.jp (Y.B.); 3 Department of Systems Pharmacology, Mie University Graduate School of Medicine, Tsu, Mie 514-8507, Japan; tanaka@doc.medic.mie-u.ac.jp

**Keywords:** neurogenesis, neurobehavior, zebrafish, comparative transcriptome analysis, gene coexpression network

## Abstract

Neurogenesis is the process by which undifferentiated progenitor cells develop into mature and functional neurons. Defects in neurogenesis are associated with neurodevelopmental and neuropsychiatric disorders; therefore, elucidating the molecular mechanisms underlying neurogenesis can advance our understanding of the pathophysiology of these disorders and facilitate the discovery of novel therapeutic targets. In this study, we performed a comparative transcriptomic analysis to identify common targets of the proneural transcription factors Neurog1/2 and Ascl1 during neurogenesis of human and mouse stem cells. We successfully identified *C3orf70* as a novel common target gene of Neurog1/2 and Ascl1 during neurogenesis. Using in situ hybridization, we demonstrated that *c3orf70a* and *c3orf70b*, two orthologs of *C3orf70*, were expressed in the midbrain and hindbrain of zebrafish larvae. We generated *c3orf70* knockout zebrafish using CRISPR/Cas9 technology and demonstrated that loss of *c3orf70* resulted in significantly decreased expression of the mature neuron markers *elavl3* and *eno2*. We also found that expression of *irx3b*, a zebrafish ortholog of *IRX3* and a midbrain/hindbrain marker, was significantly reduced in *c3orf70* knockout zebrafish. Finally, we demonstrated that neurobehaviors related to circadian rhythm and altered light–dark conditions were significantly impaired in *c3orf70* knockout zebrafish. These results suggest that C3orf70 is involved in neural and neurobehavioral development and that defects in C3orf70 may be associated with midbrain/hindbrain-related neurodevelopmental and neuropsychiatric disorders.

## 1. Introduction

Neurogenesis is the process by which undifferentiated progenitor cells, including embryonic and induced pluripotent stem cells, or previously differentiated somatic cells, develop into mature and functional neurons. Defects in neurogenesis are associated with neurodevelopmental disorders, such as autism [1,2,3] and intellectual disability [4,5], as well as adolescent and adult-onset neuropsychiatric disorders, such as major depression [6,7] and schizophrenia [2,8]. Therefore, elucidating the molecular mechanisms underlying neurogenesis can advance our understanding of the pathophysiology of these neurodevelopmental and neuropsychiatric disorders and may lead to the discovery of novel therapeutic targets.

Neurogenesis is a multi-step process involving (i) patterning of cells with neurogenic potential, which can be spread over the entire neuroectoderm or restricted to a particular domain; (ii) patterning of neural progenitors that arise within the neuroectoderm; (iii) asymmetric cell division of neural progenitor cells, which renews progenitor cells and produces a daughter cell that differentiates into a neuron or an intermediate progenitor; and (iv) movement of neural progenitors, such as integration within the surface neuroepithelium and internalization via delamination, ingression, or invagination [9].

Proneural proteins are a small group of basic-loop-helix (bHLH) transcription factors that include Achaete-scute family bHLH transcription factor 1 (Ascl1) and Neurogenin 1 and 2 (Neurog1/2). Expression of these proneural proteins is required to confer a neural fate on progenitor cells in the developing nervous system [10,11]. For example, overexpression of Ascl1 in mouse embryonic stem cells was shown to stimulate the production of neurons expressing a variety of neuronal markers, including pan-neural markers, such as neuronal class III β-tubulin (Tuj1) and microtubule associated protein 2 (MAP2); dopaminergic neuron markers, such as tyrosine hydroxylase and dopamine transporter; and motor neuron markers, such as islet 1 and 2 and the inhibitory neurotransmitter GABA [12]. Overexpression of Neurog1/2 in human induced pluripotent stem cells was also shown to stimulate the production of neurons expressing MAP2, vesicular glutamate transporter 1, and choline acetyltransferase [13]. However, downstream neuronal genes regulated by these proneural transcription factors during the initial stages of neurogenesis have not been fully elucidated. We hypothesized that Ascl1 and Neurog1/2 might regulate novel common targets in this process. To test this, we performed a comparative transcriptome analysis using publicly available datasets to analyze genes expressed downstream of Ascl1 and Neurog1/2 in human and mouse embryonic stem cells [12,13]. We then tested the function of one of the identified Ascl1 and Neurog1/2 common target genes, *C3orf70*, by examining the neural/neurobehavioral consequences of *c3orf70* gene knockout in zebrafish.

## 2. Results

### 2.1. Comparative Transcriptome Analysis Reveals Common Target Genes of Neurog1/2 and Ascl1 in Stem Cells

**Figure 1:**
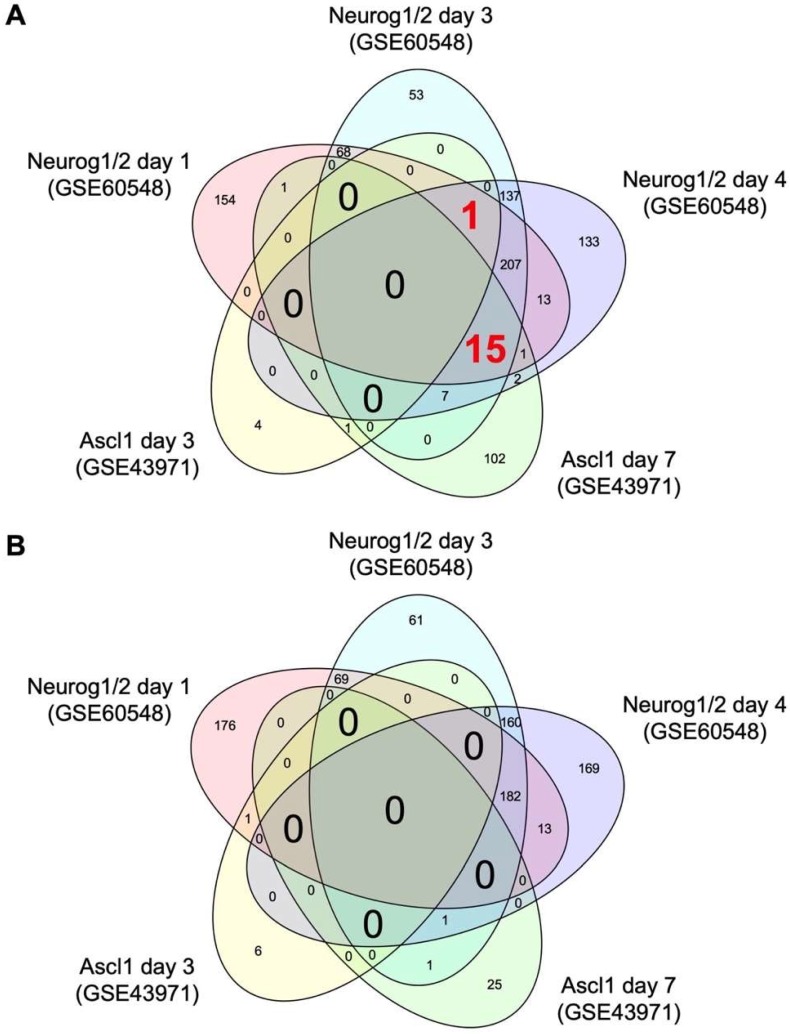
Venn diagrams of the number of differentially expressed genes regulated by Neurog1/2 and Ascl1. Transcriptome data of stem cells with and without overexpression of Neurog1/2 (GSE60548) or Ascl1 (GSE43971) were downloaded from a public database. Genes differentially expressed in stem cells on days 1, 3, and 4 post-induction of Neurog1/2 overexpression versus control cells, or on days 3 and 7 post-induction of Ascl1 overexpression versus control cells were identified using a false discovery rate threshold of 10%. The number of genes increased (**A**) and decreased (**B**) by Neurog1/2 or Ascl1 in each group and the overlap between groups are shown.

To identify common targets of Neurog1/2 and Ascl1 in stem cells, we downloaded transcriptome datasets from human stem cells with and without overexpression of Neurog1/2 (GSE60548) [13] and from mouse stem cells with and without overexpression of Ascl1 (GSE43971) [12] from the Gene Expression Omnibus [14]. Using these data, we identified differentially expressed genes (DEGs) regulated by Neurog1/2 or Ascl1 using a false discovery rate threshold of 10%. Venn diagrams of the numbers of DEGs in these datasets are shown in Figure 1. One gene, *C3orf70,* was induced at days 1, 3, and 4 after induction of Neurog1/2 overexpression and at day 3, but not day 7, after induction of Ascl1 overexpression (Table 1). Fifteen additional genes were induced by Neurog1/2 at days 1, 3, and 4, and by Ascl1 at day 7, but none of them were induced by Ascl1 at day 3 (Table 1). No genes were commonly downregulated by both Neurog1/2 and Ascl1 on the same days (Table 1). 

We next evaluated whether the 16 putative common target genes contained potential Neurog1/2 and Ascl1 binding sites by in silico analysis with Enrichr [15]. Indeed, 13 of the 16 genes contained potential binding sites for Ascl1, Neurog1, and/or Neurog2 in their promoters (Supplemental Table S1). Two other genes, cholinergic receptor nicotinic alpha 3 (*Chrna3*), and roundabout, axon guidance receptor homolog 2 (*Robo2*), lacked putative bindings sites for Ascl1, Neurog1, and/or Neurog2, but contained one for Neurod4, a bHLH proneural transcription factor that functions downstream of Neurog2 [16] (Supplemental Table S1). In addition, Enrichr-based functional analysis of 16 genes revealed significant enrichment of functions related to neuronal development (Supplemental Table S2), supporting a role for the putative Neurog1/2 and Ascl1 target genes in neuronal development.

Expression of *C3orf70,* one of the 16 genes identified as a common targets of Neurog1/2 and Ascl1, was previously shown to increase during Neurog2 and Ascl1-induced neuronal differentiation of P19 embryonic carcinoma cells [17]. To extend the Enrichr analysis, we used JASPAR, an open-access database of transcription factor-binding profiles [18], to investigate putative binding sites for Neurog2 and Ascl1 in the *C3orf70* sequence upstream of the transcription start site. This analysis confirmed the existence of binding sites in the human, mouse, and zebrafish *C3orf70* sequences (Supplemental Figure S1). Collectively, these in silico analyses support the results of the comparative transcriptome analysis and suggest that *C3orf70* is a conserved common target of Neurog2 and Ascl1 and has a potential role in neurogenesis.

### 2.2. Zebrafish Orthologs of C3orf70 Are Expressed in the Larval Midbrain and Hindbrain 

The zebrafish genome is known to contain two orthologs of *C3orf70*; *c3orf70a* and *c3orf70b*, but to our knowledge, no studies have investigated their expression and function. We first amplified the *c3orf70a* and *c3orf70b* open reading frames using cDNA reverse-transcribed from zebrafish embryo RNA and compared the sequences with those in the NCBI Reference Sequence Database (NM_001126467 and NM_001089454). A single nucleotide difference was detected between the cDNAs and the reference sequences for both *c3orf70a* and *c3orf70b* (Supplemental Figures S2 and S3), but the inferred amino acid sequences and reference sequences were identical (Supplemental Figure S4). 

**Figure 2:**
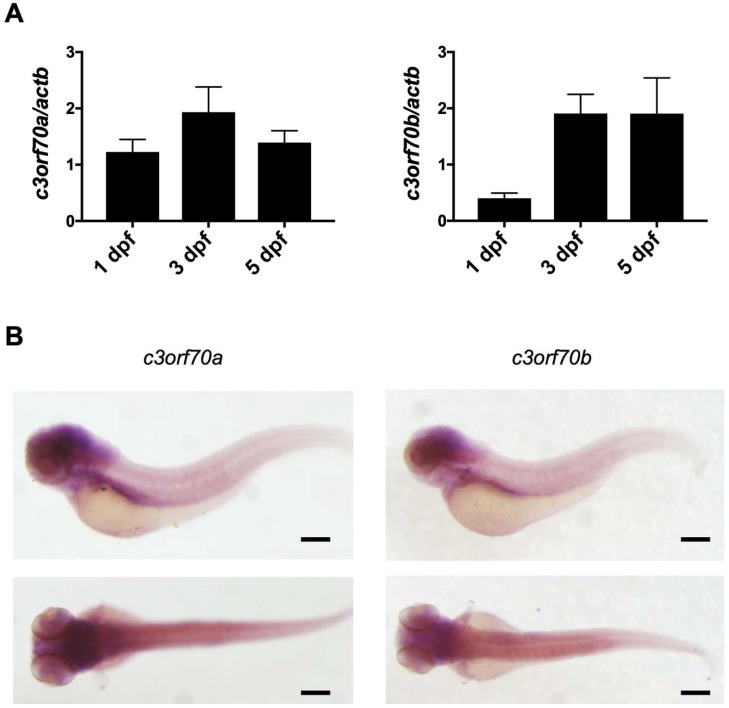
Expression of *c3orf70a* and *c3orf70b* in zebrafish. (**A**) qPCR analysis of *c3orf70a* and *c3orf70b* expression in zebrafish at 1, 3, and 5 dpf. Data are presented as the mean ± SEM of n = 3 relative to *actb* mRNA. (**B**) Whole-mount in situ hybridization of *c3orf70a* and *c3orf70b* expression in zebrafish at 3 dpf. Representative images of the lateral and dorsal views are shown. Scale bars, 200 μm.

We then examined the expression of *c3orf70a* and *c3orf70b* in zebrafish using quantitative polymerase chain reaction (qPCR) and whole-mount in situ hybridization. qPCR analysis revealed increased expression of *c3orf70a* and *c3orf70b* at 3 dpf compared with 1 dpf (Figure 2A), and whole-mount in situ hybridization revealed high expression of both *c3orf70a* and *c3orf70b* in the gut, myotomes, and brain, especially the midbrain and hindbrain, at 3 dpf (Figure 2B). Notably, the expression pattern of *c3orf70a* and *c3orf70b* were very similar, suggesting that the two genes may have redundant functions in these tissues. 

### 2.3. Generation of c3orf70-KO Zebrafish

**Figure 3:**
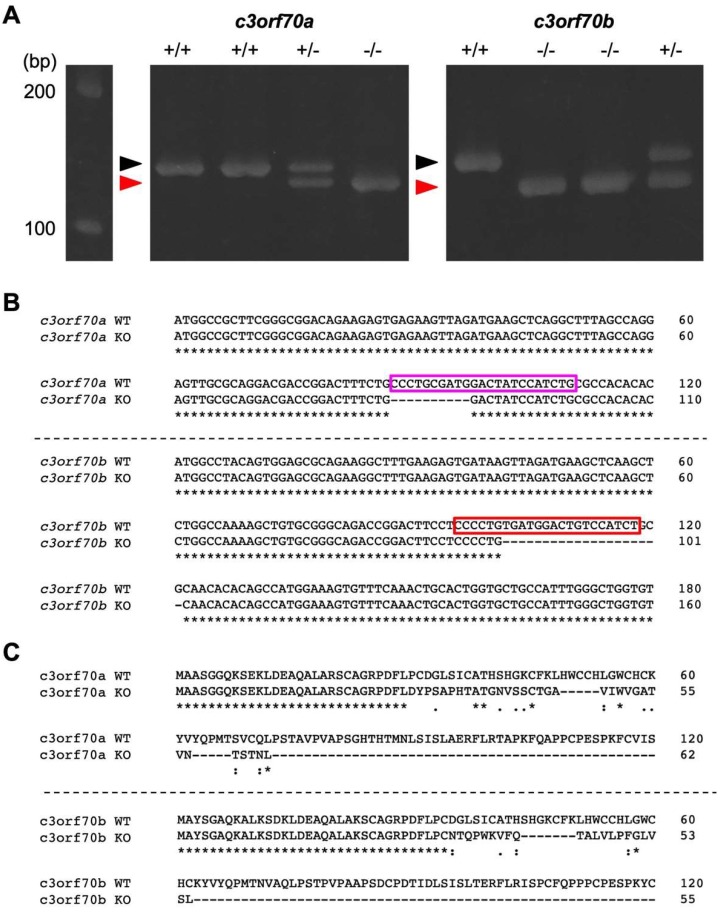
Generation of c3orf70-KO zebrafish. (**A**) Heteroduplex mobility assay of PCR products after amplification of the crRNA-targeted genomic region of *c3orf70a* and *c3orf70b*. Genomic DNA was extracted from c3orf70 wild-type (WT) and c3orf70-KO zebrafish and subjected to PCR to amplify short fragments of the *c3orf70a* and *c3orf70b* genes, including their crRNA target sites. The PCR products were electrophoresed in a 10% acrylamide gel. The positions of the expected homoduplexes for WT and KO zebrafish are indicated by black and red arrowheads, respectively. (**B**) Nucleotide sequence alignment of *c3orf70a* and *c3orf70b* genes from WT and KO zebrafish. The recognition sites of crRNA targeting *c3orf70a* and *c3orf70b,* including the PAM sequences, are shown in magenta and red boxes, respectively. (**C**) Alignment of amino acid sequences inferred from cDNA of c3orf70a and c3orf70b from WT and KO zebrafish.

To characterize the function of *c3orf70*, we generated *c3orf70a* and *c3orf70b* double knockout zebrafish (hereafter referred to as c3orf70-KO) using CRISPR/Cas9. We designed crRNAs to target these genes and injected them with tracRNA and Cas9 proteins into zebrafish embryos. After several rounds of genetic selection, we established c3orf70-KO zebrafish in which 10 and 20 bp were deleted in the *c3orf70a* and *c3orf70b* genes (Figure 3A,B), causing frame shifts that generated premature stop codons (Figure 3C). The c3orf70-KO zebrafish were viable and fertile.

### 2.4. Impaired Neuronal Marker Expression in c3orf70-KO Zebrafish

**Figure 4:**
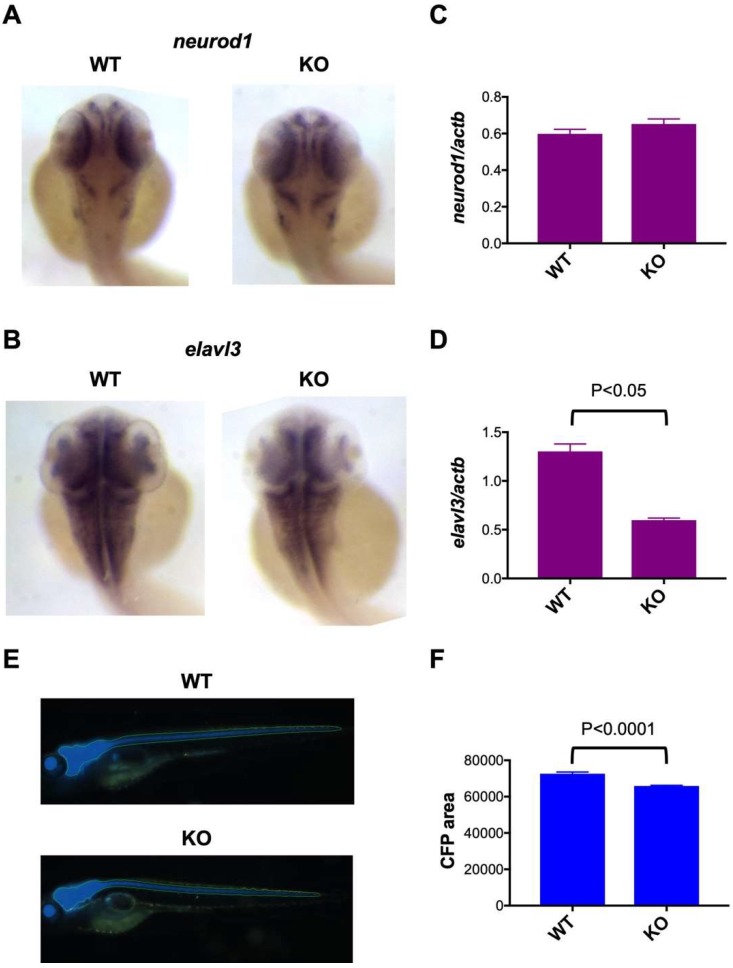
Impaired neuronal marker expression in c3orf70-KO zebrafish. (**A**–**D**) Whole-mount in situ hybridization (**A**,**B**) and qPCR (**C**,**D**) of *neurod1* (**A**,**C**) and *elavl3* (**B**,**D**) expression in c3orf70 WT and KO zebrafish at 2 dpf. Data are presented as the mean ± SEM of *n* = 4 for both WT and KO. (**E**,**F**) Representative images (**E**) and quantification of fluorescence (**F**) in Tg (eno2: Cerulean) c3orf70-WT or KO zebrafish at 5 dpf. Data are presented as the mean ± SEM of *n* = 60 and 62 for WT and KO groups, respectively.

To analyze the functional role of *c3orf70* in neuronal development, we compared the expression of the neuronal markers neuronal differentiation 1 (*neurod1*), ELAV-like neuron-specific RNA binding protein 3 (*elavl3*), and enolase 2 (*eno2*) in c3orf70-WT and KO zebrafish. Neurod1 is a bHLH transcription factor commonly used as a proneural marker [19], Elavl3 regulates alternative splicing of several pre-mRNAs and has been used as a pan-neuronal marker [20], and Eno2 (enolase) is a glycolytic enzyme highly expressed in mature neurons [21]. Whole-mount in situ hybridization and qPCR analysis revealed that *neurod1* expression was not significantly different in c3orf70-WT and KO zebrafish (Figure 4A,C), whereas *elavl3* was present at significantly lower levels in c3orf70-KO compared with the WT zebrafish (Figure 4B,D). In vivo imaging of transgenic zebrafish in which the fluorescent protein cerulean was expressed in mature neurons under the control of the *eno2* promoter (Figure 4E) revealed a significant reduction in fluorescence in c3orf70-KO compared with WT zebrafish (Figure 4F). qPCR analysis of *eno2* revealed the same trend (data not shown). These results suggest that c3orf70 may regulate neuronal differentiation and maturation via *elavl3* and *eno2*. 

### 2.5. WGCNA Identifies IRX3 as a Gene Coexpressed with C3orf70 During Neurogenesis

**Figure 5:**
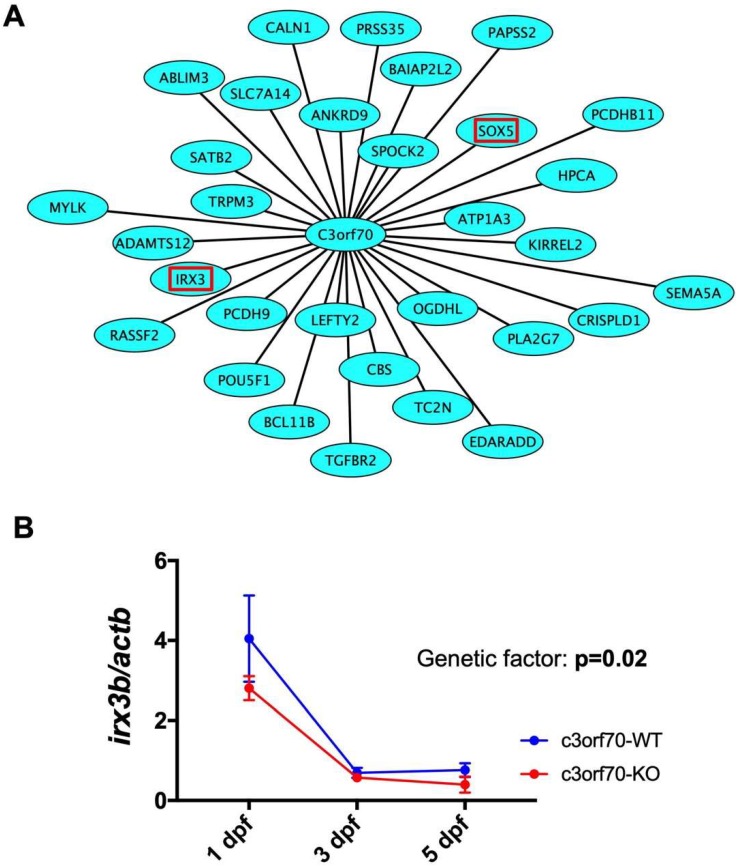
Weighted gene coexpression network analysis (WGCNA) identifies *IRX3* as a gene coexpressed with C3orf70 during neurogenesis. (**A**) Schematic showing the 31 genes coexpressed with *C3orf70* in human stem cells overexpressing Neurog1/2 (GSE60548) [13] and mouse stem cells overexpressing Ascl1 (GSE43971) [12], as identified by WGCNA. *IRX3* and *SOX5* are outlined in red. (**B**) qPCR analysis of *irx3b* expression in c3orf70-WT and KO zebrafish. Data are presented as the mean ± SEM of *n* = 3.

To analyze the potential molecular mechanisms by which *c3orf70* KO may impair neurogenesis in zebrafish, we performed weighted gene coexpression network analysis (WGCNA), which organizes transcriptomic data into networks based on gene coexpression to elucidate novel connections between genes [22,23]. Using WGCNA, we analyzed the transcriptome data of human stem cells with and without induction of Neurog1/2 overexpression (GSE60548) [13] and of mouse stem cells with and without induction of Ascl1 overexpression (GSE43971) [12] and identified 1073 and 546 genes, respectively, coexpressed with *C3orf70* during neurogenesis. Of these, 31 genes, including the midbrain and hindbrain marker *IRX3* [24,25], were identified as commonly coexpressed with *C3orf70* after induction of both Neurog1/2 and Ascl1 (Figure 5A). qPCR analysis showed that expression of *irx3b*, a zebrafish ortholog of *IRX3*, was significantly reduced in c3orf70-KO zebrafish at 3 and 5 dpf (Figure 5B). These results suggest that impaired neurogenesis in c3orf70-KO zebrafish might be associated with disturbances in *c3orf70*-coexpressed genes such as *irx3b*.

### 2.6. Circadian Behavioral Responses Are Impaired in c3orf70-KO Zebrafish

**Figure 6:**
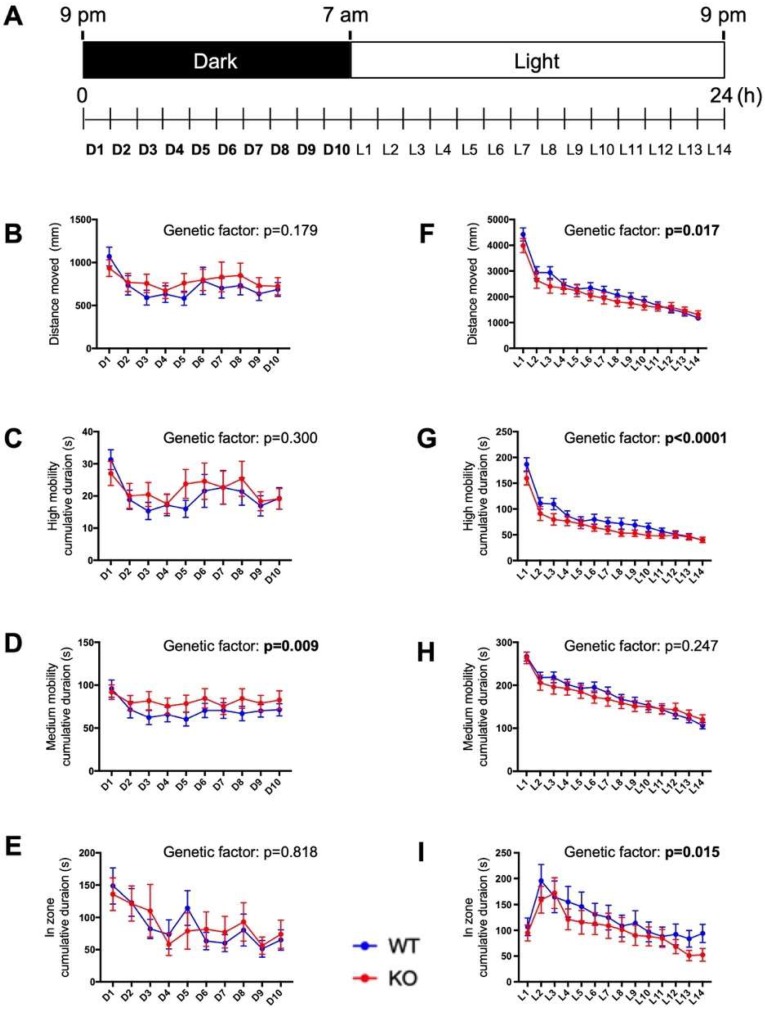
Impaired circadian behavior in c3orf70-KO zebrafish. (**A**) Overview of the circadian behavioral analysis. Behavior was assessed on 7 to 8 dpf using four endpoints: distance moved (**B**,**F**), cumulative duration at high mobility (**C**,**G**) and medium mobility (**D**,**H**), and cumulative duration in the center zone (**E**,**I**). Behavior during the dark and light periods is shown in (**B**–**E**) and (**F**–**I)**, respectively. Data are presented as the mean ± SEM of *n* = 40 for WT and *n* = 41 for KO.

Because the midbrain region, where zebrafish *c3orf70* orthologs are highly expressed, is one of the most important regions in controlling sleep–wake systems [26], we next compared the circadian behavior of c3orf70-WT and KO zebrafish by analyzing the distance moved and the time spent in movement during periods of light and dark (Figure 6A). During the dark period (21:00–07:00), the cumulative time spent at medium levels of mobility was significantly longer for c3orf70-KO compared with WT zebrafish (Figure 6D), whereas the total distance moved, the cumulative duration at high mobility, and cumulative duration in the center zone of the well (i.e., relative inactivity) were not significantly different between the WT and KO zebrafish (Figure 6B,C,E). During the light period, however (07:00–21:00), c3orf70-KO zebrafish displayed a significantly decreased total distance moved, cumulative duration at high mobility, and cumulative duration in the center zone compared with the WT zebrafish (Figure 6F,G,I). These results suggest that *c3orf70* deletion impaired the resting activity of zebrafish during the night and their explorative activity during the day.

### 2.7. Behavioral Responses to Alternating Light–Dark Cycles Is Impaired in c3orf70-KO Zebrafish

**Figure 7:**
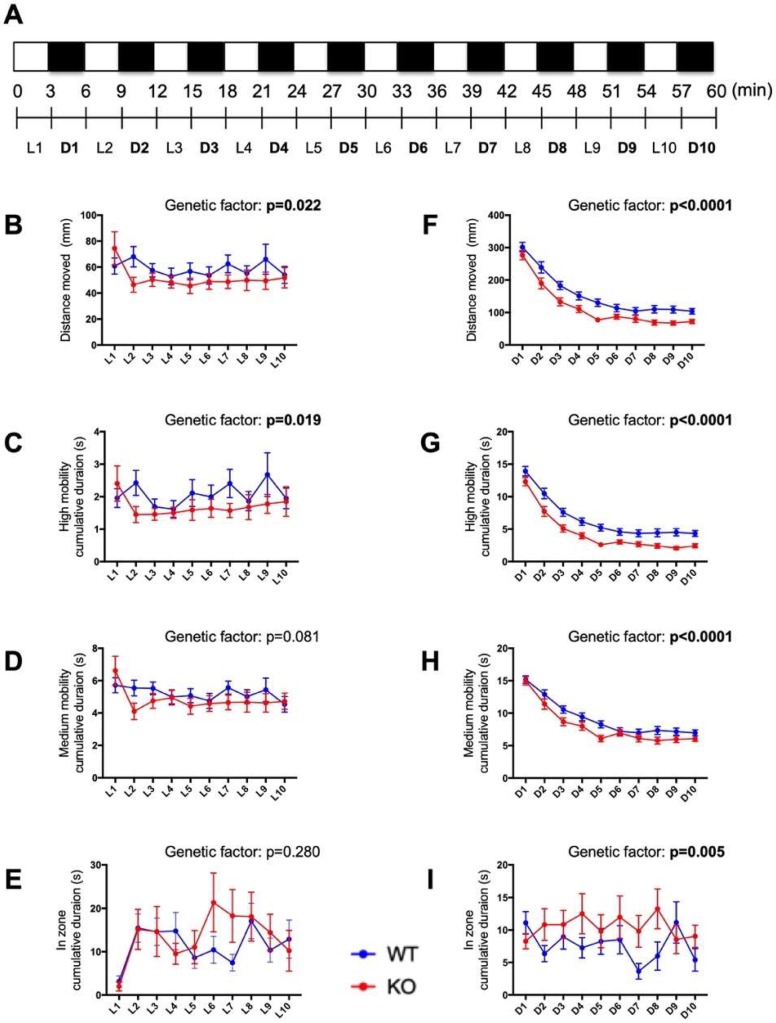
Impaired behavioral responses to light–dark cycling in c3orf70-KO zebrafish. (**A**) Overview of the behavioral analysis of the response to 3-min cycling between light and dark conditions. Behavior in c3orf70-WT or KO was assessed as: distance moved (**B**,**F**), cumulative duration at high mobility (**C**,**G**) and medium mobility (**D**,**H**), and cumulative duration in the center zone (**E**,**I**). Behavior during dark and light periods is shown in (**B**–**E**) and (**F**–**I**), respectively. Data are presented as the mean ± SEM of *n* = 40 for WT and *n* = 41 for KO.

Previous studies have employed repeated cycling between relatively short periods of light and dark conditions (several minutes per condition) to examine the motor function and emotional behavior of zebrafish [27,28]. The midbrain contains several structures important in the regulation of motor function and emotion [29]. Therefore, we compared the behavior of c3orf70-WT and KO zebrafish in response to 3-min alternating intervals of light and dark. The total distance moved and cumulative duration at high mobility were both significantly decreased in c3orf70-KO zebrafish during both the light and dark periods (Figure 7B,C,F,G), whereas the cumulative duration in the center zone was significantly increased by c3orf70 KO only during the dark periods (Figure 7I). These results suggest that attention and/or normal anxiety levels might be disrupted in c3orf70-KO zebrafish. 

## 3. Discussion

In this study, we identified *C3orf70* as a common target gene of Neurog1/2 and Ascl1 in human and mouse embryonic stem cells during neurogenesis. This finding is consistent with a previous report demonstrating that C3orf70 expression is increased during Neurog2- and Ascl1-initiated neuronal differentiation of the P19 mouse embryonic carcinoma cell line [17]. In addition, we identified putative binding sites for Neurog2 and Ascl1 in the *C3orf70* promoter of humans, mice, and zebrafish. We also investigated the expression and function of *c3orf70* in zebrafish and found that it was required for normal expression of the post-mitotic and mature neuron markers *elavl3* and *eno2* and the behaviors related to circadian rhythm and changes in light–dark conditions in zebrafish larvae. Collectively, these results suggest that C3orf70 is involved in neurogenesis and neurobehavior, and that impairments in C3orf70 expression and/or function may be linked to neurodevelopmental and neuropsychiatric disorders. 

We were unable to identify any known functional motifs in the amino acid sequence of C3orf70 that might point to its potential mechanism of action in neurogenesis and neurobehavior. Therefore, we sought to identify genes coexpressed with *C3orf70* during neurogenesis. Of the 31 genes identified, two, *IRX3* and *SOX5*, were of particular interest [12,13]. *IRX3* is highly expressed in the midbrain and hindbrain of vertebrates, including zebrafish (*irx3*), and is involved in neurogenesis [24,25,30]. The midbrain is involved in regulation of circadian behavior and behavioral responses related to motor function and emotion [27,28] [26,29], which we confirmed were disrupted in c3orf70-KO zebrafish. These findings suggest that C3orf70 may regulate neurogenesis and neurobehavioral development via interactions with coexpressed genes such as *IRX3*. It is also noteworthy that both *C3orf70* and *IRX3* are genetically associated with obesity [31,32]. *SOX5* is associated with neuronal development, intellectual disability, and autism [33,34,35]. Interestingly, genetic analyses have demonstrated that C3orf70 is associated with educational attainment [36], major depressive disorder [37], and insomnia [38]. Although we could not detect a significant difference in the expression of *sox5* in c3orf70-KO and WT zebrafish using qPCR (data not shown), SOX5, similar to C3orf70, is highly expressed in the midbrain [39], suggesting a possible link in their function. Additional analyses, such as in situ hybridization, may shed light on the differential expression of *sox5* in WT and c3orf70-KO zebrafish. Moreover, further studies will be required to fully elucidate the mechanisms by which C3orf70 regulates IRX3 and SOX5 expression as well as their involvement in neurogenesis and neurobehavior. In this regard, it will be important to analyze zebrafish with single- and double-KO of *c3orf70a/c3orf70b* over many generations to clarify the function of the two genes and to exclude possible off-target effects of CRISPR/Cas9-induced mutations. Rescue experiments involving injection of mRNA encoding c3orf70a and/or c3orf70b will also be required. 

In this study, we used zebrafish as a model organism in which to analyze the function of *c3orf70*. Zebrafish have been successfully used to characterize the function of numerous genes in vivo [40,41] and to find novel therapeutics for various diseases [42,43]. Although zebrafish and mammals display several key developmental differences, such as ex utero development and the eversion of telencephalic hemispheres in zebrafish, comparative neurogenetic and neuroanatomical analyses have revealed a high degree of conservation in neurogenesis between zebrafish and mammals [28,44,45]. Moreover, zebrafish express orthologs of many molecules that are therapeutic targets in humans [46]. Although some studies have shown differences in pharmacodynamics between zebrafish and humans [47,48], many other studies have demonstrated the utility of zebrafish to identify novel drugs and to investigate the safety of drugs in preclinical and clinical development [28,43,49,50,51]. The results of the present study suggest that c30rf70-KO zebrafish may be useful for understanding the pathophysiology of neurodevelopmental and neuropsychiatric disorders related to C3orf70 function, and to identify novel drugs to treat these disorders. It should be noted, however, that we were not able to examine the function of *c3orf70a* and *c3orf70b* separately in this study, and further experiments will be required to clarify their individual roles.

In conclusion, we performed a comparative transcriptomic analysis of neurogenesis of human and mouse stem cells and identified *C3orf70* as a novel common target of Neurog1/2 and Ascl1. Using zebrafish, we demonstrated that *c3orf70* is involved in neurogenesis and neurobehavior. These results suggest that impairments in C3orf70 might be related to human neurodevelopmental and neuropsychiatric disorders, and they provide a strong rationale for further characterization of C3orf70 as a potential therapeutic target. 

## 4. Materials and Methods

### 4.1. Ethics Statement

Mie University Institutional Animal Care and Use Committee guidelines state that no approval is required for experiments using zebrafish. However, all animal experiments described in this manuscript conform to the ethical guidelines established by the Institutional Animal Care and Use Committee at Mie University.

### 4.2. Comparative Transcriptome Analysis

To compare DEGs caused by the activation of Neurog1/2 or Ascl1 in human or mouse stem cells, respectively, we used two transcriptome datasets deposited in the Gene Expression Omnibus (GEO) [52]. The normalized transcriptome analysis data of human (GSE60548) [13] and mouse stem cells (GSE43971) [12] were downloaded from GEO and subjected to “RankProd” [53] in Bioconductor [54] to identify the DEGs using a false discovery rate threshold of 10%. The gene symbols of the DEGs in mouse stem cells were converted to the human orthologs using the Life Science Knowledge Bank (World Fusion, Tokyo, Japan). 

### 4.3. Bioinformatic Analysis of Common DEGs

We used Enrichr [15] to identify transcription factors that potentially regulate the identified DEGs and to examine the putative biological functions enriched for each common DEG. Briefly, a list of common DEGs (Table 1) was subjected to Enrichr analysis, and factors/processes returned in the “Enrichr Submissions TF-Gene Cooccurrence” (adjusted *p* < 1 × 10^−6^) and “GO Biological Process 2018” (adjusted *p* < 0.05) were identified as transcription factors potentially regulating the common DEGs (Supplemental Table S1) and biological functions enriched in the common DEGs (Supplemental Table S2), respectively. JASPAR [18] was used to identify the putative binding sites for Neurog2 and Ascl1 transcription factors in a 3000 bp sequence upstream of the transcription start site in human, mouse, and zebrafish *C3orf70*. 

### 4.4. Zebrafish Husbandry

We used the Tg (*eno2*: Cerulean) zebrafish line [55] to derive the strains generated here. Zebrafish were maintained as described previously [50]. Briefly, zebrafish were raised at 28.5 ± 0.5 °C with a 14 h/10 h light/dark cycle. Embryos were obtained via natural mating and cultured in 0.3× Danieau’s solution (19.3 mM NaCl, 0.23 mM KCl, 0.13 mM MgSO_4_, 0.2 mM Ca(NO_3_)_2_, 1.7 mM HEPES, pH 7.2). 

### 4.5. Generation of c3orf70-KO Zebrafish 

C3orf70-KO zebrafish were generated according to methods described previously [56,57], with some modifications. Briefly, CRISPR RNA (crRNA) targeting the *c3orf70a* (*si:dkey-22o12.2*) or *c3orf70b* (*zgc:162707*) genes and trans-activating crRNA (tracrRNA) [58] were obtained from FASMAC (Kanagawa, Japan) (sequences shown in Supplemental Table S3). Recombinant Cas9 protein was obtained from Toolgen (Seoul, Korea). crRNA, tracrRNA, and Cas9 protein were dissolved in sterilized water (1000 ng/µL) and stored at −80 °C until required. For microinjection, crRNAs, tracrRNA, Cas9 protein, and a lissamine-labeled control morpholino with no known target gene (Gene Tools, Philomath, OR, USA) were mixed in Diethylpyrocarbonate ()-treated water to final concentrations of 200 ng/µL (100 ng/µL each), 100 ng/µL, 400 ng/µL, and 50 nM, respectively. The solution was injected into 1-cell-stage zebrafish embryos derived from the Tg (eno2: cerulean) line. At 4 months post-fertilization, genomic DNA was extracted from the fins of F0 zebrafish and used to detect CRISPR/Cas9-induced mutations according to previous reports [59,60] with some modifications as follows. A short fragment of the *c3orf70a* or *c3orf70b* gene encompassing the target sites was amplified from genomic DNA using the primers shown in Supplemental Table S3. Three-step PCR was carried out using 40 cycles of 94 °C for 30 s, 60 °C for 30 s, and 68 °C for 30 s. The PCR products were electrophoresed in 10% polyacrylamide gels as described previously [59,60] and F0 fish in which the CRISPR/Cas9-induced mutation was present were crossed with the Tg(*eno2*: Cerulean) zebrafish line to obtain F1 progeny (*c3orf70a+/-*:*c3orf70b+/+* or *c3orf70a+/+*:*c3orf70b+/-*). The F1 generation was reared and screened for the presence of the mutation by PCR, as described above. F1 female and male hetero-KO zebrafish harboring the same mutations in the *c3orf70a* or *c3orf70b* gene were crossed to obtain F2 progeny (*c3orf70a-/-*:*c3orf70b+/*+, *c3orf70a+/+*:*c3orf70b-/*-, and *c3orf70a+/-*:*c3orf70b+/*-). The PCR product corresponding to the homo-KO of *c3orf70a* or *c3orf70b* was subjected to sequence analysis using ExoSAP-IT Express PCR Cleanup Reagents (Thermo Fisher, MA, USA) according to the manufacturer’s protocol. F2 fish (*c3orf70a+/-*:*c3orf70b+/*-) with a homozygous genotype (validated to have the frame shift mutation, as shown in Figure 2) were used to generate F3 progeny (*c3orf70a-/-*:*c3orf70b-/-*). The F3 generation was reared and crossed to obtain F4 progeny. The F4 generation was characterized in this study. The PCR product corresponding to the WT or homo-KO of *c3orf70a* or *c3orf70b* cDNA was also subjected to sequence analysis as shown above.

### 4.6. Whole-Mount In Situ Hybridization of Neuronal Markers

Whole-mount in situ hybridization was performed as described previously [61,62] with some modifications. Briefly, cDNA fragments of *c3orf70a*, *c3orf70b, neurod1,* and *elavl3* were amplified using the primers shown in Supplemental Table S3. The PCR products were cloned into the pGEM-T vector (Promega, WI, USA) and sequence analysis was performed to verify insertion in the correct orientation. Antisense probes were synthesized using the DIG RNA Labeling Kit (Sigma-Aldrich, MO, USA). c3orf70-KO or WT Tg (eno2: cerulean) zebrafish were fixed at 2 dpf (*neurod1* and *elavl3*) and 3 dpf (*c3orf70a* and *c3orf70b*) for whole-mount in situ hybridization.

### 4.7. qPCR Analysis

qPCR analysis was performed as described previously [42,60] with some modifications. Briefly, total RNA was extracted from Tg (eno2: cerulean) zebrafish using a Nucleospin RNA XS kit (Takara, Kyoto, Japan) according to the manufacturer’s protocol. cDNA was generated using a ReverTra Ace qPCR RT Kit (Toyobo). qPCR was performed using an ABI Prism 7300 PCR system (Life Technologies, Carlsbad, CA, USA) with THUNDERBIRD SYBR qPCR Mix (Toyobo). The thermal cycling conditions were: 95 °C for 1 min, followed by 40 cycles of 95 °C for 15 s, 60 °C for 15 s, and 72 °C for 45 s. mRNA expression of *neurod1, elavl3, eno2* and *irx3b* was normalized to that of β-actin (*actb*) to correct for variability in the initial template concentration and reverse transcription efficiency. The primer sequences are shown in Supplemental Table S3.

### 4.8. Weighted Gene Coexpression Network Analysis

The coefficient of variation (CV) of the normalized probe intensity for each gene in the GSE60548 [13] and GSE43971 [12] transcriptome datasets was calculated, and genes were sorted in descending order by CV. The top 3000 genes were subjected to WGCNA [63] in Bioconductor [54]. *C3orf70* was included in the top 3000 gene lists from both transcriptome datasets. Seven and 13 modules were classified based on coexpression between the top 3000 genes of GSE60548 and GSE43971, respectively. *C3orf70* was included in the turquoise modules in both datasets. Genes coexpressed with *C3orf70* in each turquoise module were selected using thresholds of 0.1. The relationships were analyzed in Cytoscape [64] to identify and draw the common network between *C3orf70* and the coexpressed genes in both datasets.

### 4.9. In Vivo Imaging of Tg (eno2: Cerulean) Zebrafish

At 5 dpf, c3orf70-KO or WT Tg (eno2: cerulean) zebrafish were anesthetized with 2-phenoxyethanol and placed in a 96-well imaging plate (ZF plate, Hashimoto Electric Industry, Mie, Japan). In vivo imaging and quantitative analysis of the cerulean fluorescence signal was performed using ImageXpress Micro with customized programs (Molecular Device, Sunnyvale, CA, USA). Brain and spinal cord regions expressing *eno2* promoter-driven cerulean fluorescence above a defined threshold were automatically detected and the areas were quantified.

### 4.10. Behavioral Analysis

At 7 dpf, zebrafish were placed individually into the wells of a 48-well plate (10 mm radius, 800 µL of 0.3× Danieau’s solution) at 20:00 (n = 20–24 for each group). The 48-well plate was placed in an incubator at 28.5 °C with constant light (255 lx) between 20:00–21:00 and then placed in a DanioVision system (Noldus, Wageningen, The Netherlands), which analyzes circadian behavior. The 48-well plate in DanioVision was kept at 28.5 °C in the dark between 21:00 and 07:00 and illuminated from below with white light (255 lx) between 07:00 and 21:00. After circadian behavior analysis, behavioral responses to light–dark changes were analyzed using 10 cycles of alternating light and dark, consisting of illumination with white light (255 lx) for 3 min followed by no light for 3 min. Zebrafish behavior was monitored in each well by DanioVision at a resolution of 1024 × 768 pixels and 25 frames per second. Two independent experiments were performed. All recorded video images were subjected to EthoVision XT11 (Noldus) to measure total distance moved, cumulative time spent at high or medium mobility, and cumulative time spent in the center zone (circle of 2 mm radius) of the well. Mobility was calculated by comparing every pixel in the current and previous images. If all pixels were identical, zero mobility was recorded. If all pixels were different, 100% mobility was recorded. In this study, we defined medium and high mobility as 35–65% and 65–95% difference in pixels, respectively.

### 4.11. Statistical analysis

Statistical analysis was performed using Prism 7 (GraphPad, La Jolla, CA, USA). Mann–Whitney tests were performed to assess differences between neuronal marker expression, and two-way ANOVA was performed to assess zebrafish behavior and *irx3b* expression. Data are presented as the mean ±SEM of the indicated number of zebrafish.

## Figures and Tables

**Table 1:** Differentially expressed genes regulated by Neugog1/2 and Ascl1.

Symbol	Neurog1/2 Day 1		Neurog1/2 Day 3		Neurog1/2 Day 4		Ascl1 Day 3		Ascl1 Day 7	
	FC	FDR	FC	FDR	FC	FDR	FC	FDR	FC	FDR
*C3orf70*	8.13	2.761 × 10^−2^	24.41	1.11 × 10^−2^	23.27	1.91 × 10^−2^	1.54	3.67 × 10^−2^		
*CHGB*	4.80	7.97 × 10^−2^	12.25	4.08 × 10^−2^	14.02	4.98 × 10^−2^			1.89	2.21 × 10^−2^
*CHRNA3*	26.20	2.17 × 10^−3^	23.90	1.13 × 10^−2^	39.49	7.76 × 10^−3^			1.72	3.30 × 10^−2^
*DCX*	11.89	1.22 × 10^−2^	38.49	4.20 × 10^−3^	102.50	9.74 × 10^−4^			2.91	0.0000
*EBF2*	90.33	3.33 × 10^−5^	24.69	1.08 × 10^−2^	37.51	8.50 × 10^−3^			1.84	2.51 × 10^−2^
*ELAVL3*	100.60	9.81 × 10^−6^	360.60	3.26 × 10^−6^	509.80	8.26 × 10^−6^			1.50	7.38 × 10^−2^
*ELAVL4*	10.17	1.78 × 10^−2^	100.80	5.48 × 10^−4^	172.60	2.61 × 10^−4^			1.62	4.85 × 10^−2^
*GFRA1*	40.96	5.87 × 10^−4^	54.30	2.23 × 10^−3^	120.00	6.45 × 10^−4^			1.65	4.44 × 10^−2^
*INSM1*	222.40	1.23 × 10^−7^	290.70	1.35 × 10^−5^	461.80	1.47 × 10^−5^			1.50	7.42 × 10^−2^
*ISL1*	24.59	2.51 × 10^−3^	97.31	5.67 × 10^−4^	227.50	1.15 × 10^−4^			1.59	5.34 × 10^−2^
*MDGA1*	110.60	2.84 × 10^−6^	291.10	3.54 × 10^−6^	224.50	4.52 × 10^−5^			1.54	6.46 × 10^−2^
*MYT1*	5.63	5.69 × 10^−2^	45.99	3.05 × 10^−3^	74.14	2.16 × 10^−3^			2.09	1.05 × 10^−2^
*ONECUT2*	11.57	1.27 × 10^−2^	85.82	7.27 × 10^−4^	112.50	7.48 × 10^−4^			1.48	8.22 × 10^−2^
*PCDH9*	8.05	2.91 × 10^−2^	79.33	9.67 × 10^−4^	94.84	1.24 × 10^−3^			1.49	7.73 × 10^−2^
*POU3F2*	38.74	6.63 × 10^−4^	256.10	2.35 × 10^−5^	189.20	1.85 × 10^−4^			1.70	3.55 × 10^−2^
*ROBO2*	12.58	1.05 × 10^−2^	27.02	9.23 × 10^−3^	73.36	2.16 × 10^−3^			1.49	7.67 × 10^−2^

## Supplementary Materials

The following are available online at https://www.mdpi.com/1424-8247/12/4/156/s1, Figure S1: Comparison of the putative binding sites for Neurog2 and Ascl1 in the promoter sequence of C3orf70. Figure S2: Comparison of the nucleotide sequences of c3orf70a open reading frame determined in this study and human sequence NM_001126467 in the NCBI Reference Sequence Database. Figure S3: Comparison of the nucleotide sequences of c3orf70b open reading frame determined in this study and NM_001089454 in the NCBI Reference Sequence Database. Figure S4: Comparison of the inferred amino acid sequences of c3orf70a and c3orf70b open reading frames obtained in this study and NCBI reference sequences NM_001126467 and NM_001089454. Table S1: Transcription factors enriched in the promoters of differentially expressed genes regulated by Neurog1/2 and Ascl1. Table S2: Biological process enriched in the differentially expressed genes regulated by Neurog1/2 and Ascl1. Table S3: crRNA, tracrRNA, and PCR primer nucleotide sequences used for this study.
