# Supplementary material for: C3orf70 Is Involved in Neural and Neurobehavioral Development
Source: Pharmaceuticals (Basel). 2019 Oct 16;12(4):156. doi: 10.3390/ph12040156 (PMC6958487; doi:10.3390/ph12040156)
Supplement: Supplementary file 1 [file pharmaceuticals-12-00156-s001.pdf]

Supplemental Table 1. Transcription factors enriched in the promoters of differentially expressed genes regulated by Neurog1/2 and Ascl1

Term	Adjusted P-value	Genes
NEUROD4	3.55E-19	*ROBO2;CHRNA3;ONECUT2;ELAVL4;EBF2;GFRA1;MYT1;POU3F2;ISL1;ELAVL3;DCX;INSM1;CHGB*
NHLH2	3.97E-17	*ROBO2;CHRNA3;ONECUT2;ELAVL4;DCX;EBF2;GFRA1;INSM1;MYT1;POU3F2;ISL1;ELAVL3*
ASCL1	3.26E-15	*PCDH9;ONECUT2;ELAVL4;DCX;GFRA1;INSM1;MYT1;POU3F2;ISL1;CHGB;ELAVL3*
NHLH1	3.26E-15	*ONECUT2;ELAVL4;DCX;EBF2;INSM1;MDGA1;MYT1;POU3F2;ISL1;CHGB;ELAVL3*
SCRT2	2.72E-13	*ROBO2;ONECUT2;ELAVL4;DCX;INSM1;MDGA1;MYT1;POU3F2;CHGB;ELAVL3*
NEUROD1	2.72E-13	*ROBO2;ONECUT2;ELAVL4;DCX;INSM1;MYT1;POU3F2;ISL1;CHGB;ELAVL3*
INSM1	1.59E-11	*ROBO2;ONECUT2;ELAVL4;DCX;MYT1;POU3F2;ISL1;CHGB;ELAVL3*
ARX	1.59E-11	*ROBO2;PCDH9;ONECUT2;DCX;EBF2;INSM1;POU3F2;ISL1;CHGB*
POU3F4	1.59E-11	*ROBO2;ONECUT2;ELAVL4;DCX;GFRA1;INSM1;MYT1;POU3F2;ISL1*
FEZF2	1.59E-11	*ROBO2;ONECUT2;ELAVL4;DCX;EBF2;GFRA1;INSM1;POU3F2;ISL1*
ISL1	5.27E-10	*ROBO2;ONECUT2;ELAVL4;EBF2;GFRA1;INSM1;POU3F2;CHGB*
FOXG1	5.27E-10	*ROBO2;PCDH9;ONECUT2;DCX;GFRA1;INSM1;POU3F2;ISL1*
LHX9	5.27E-10	*ROBO2;PCDH9;ONECUT2;EBF2;GFRA1;INSM1;POU3F2;ISL1*
SOX1	5.27E-10	*ONECUT2;ELAVL4;EBF2;GFRA1;INSM1;POU3F2;ISL1;ELAVL3*
POU3F2	5.27E-10	*ROBO2;PCDH9;ONECUT2;DCX;EBF2;GFRA1;INSM1;ISL1*
SIM1	5.27E-10	*ROBO2;PCDH9;ONECUT2;EBF2;GFRA1;INSM1;POU3F2;ISL1*
PHOX2B	5.27E-10	*ONECUT2;ELAVL4;EBF2;GFRA1;INSM1;POU3F2;ISL1;ELAVL3*
MYT1	5.27E-10	*ROBO2;ONECUT2;ELAVL4;DCX;INSM1;POU3F2;CHGB;ELAVL3*
ATOH1	5.27E-10	*ROBO2;ONECUT2;EBF2;GFRA1;INSM1;POU3F2;ISL1;CHGB*
NEUROD2	5.27E-10	*ONECUT2;ELAVL4;DCX;INSM1;MYT1;POU3F2;CHGB;ELAVL3*
ST18	5.27E-10	*ROBO2;PCDH9;ELAVL4;DCX;INSM1;MYT1;ISL1;CHGB*
DRGX	5.27E-10	*ONECUT2;ELAVL4;EBF2;GFRA1;INSM1;POU3F2;ISL1;ELAVL3*
SCRT1	5.27E-10	*ONECUT2;ELAVL4;INSM1;MDGA1;MYT1;POU3F2;CHGB;ELAVL3*
ONECUT2	1.78E-08	*ROBO2;DCX;EBF2;GFRA1;INSM1;POU3F2;ISL1*
HOXD3	1.78E-08	*ROBO2;ONECUT2;EBF2;GFRA1;INSM1;POU3F2;ISL1*
PAX2	1.78E-08	*ONECUT2;EBF2;GFRA1;INSM1;MDGA1;POU3F2;ISL1*
POU4F1	1.78E-08	*ONECUT2;DCX;EBF2;INSM1;POU3F2;ISL1;ELAVL3*
TBR1	1.78E-08	*ROBO2;ONECUT2;ELAVL4;DCX;INSM1;POU3F2;ISL1*
TFAP2B	1.78E-08	*ROBO2;ONECUT2;EBF2;GFRA1;INSM1;POU3F2;ISL1*
PAX3	1.78E-08	*ROBO2;ONECUT2;EBF2;GFRA1;INSM1;POU3F2;ISL1*
SP8	1.78E-08	*ROBO2;ONECUT2;EBF2;GFRA1;INSM1;POU3F2;ISL1*
NEUROG1	1.78E-08	*ONECUT2;EBF2;INSM1;MDGA1;MYT1;POU3F2;ISL1*
POU4F2	1.78E-08	*ONECUT2;EBF2;GFRA1;INSM1;POU3F2;ISL1;ELAVL3*
DLX6	1.78E-08	*ROBO2;ONECUT2;EBF2;GFRA1;INSM1;POU3F2;ISL1*
SALL3	1.78E-08	*ROBO2;ONECUT2;EBF2;GFRA1;INSM1;POU3F2;ISL1*
INSM2	1.78E-08	*ONECUT2;ELAVL4;EBF2;INSM1;POU3F2;ISL1;CHGB*
PAX4	1.78E-08	*ONECUT2;DCX;INSM1;MYT1;POU3F2;ISL1;CHGB*
ZIC4	1.78E-08	*ROBO2;ONECUT2;EBF2;GFRA1;INSM1;POU3F2;ISL1*
TFAP2D	1.78E-08	*ROBO2;PCDH9;ONECUT2;ELAVL4;EBF2;POU3F2;ISL1*
RFX6	1.78E-08	*ROBO2;ONECUT2;ELAVL4;DCX;INSM1;ISL1;CHGB*
BARHL1	1.78E-08	*ONECUT2;EBF2;INSM1;MYT1;POU3F2;ISL1;CHGB*
ISL2	4.26E-07	*ONECUT2;EBF2;GFRA1;INSM1;POU3F2;ISL1*
ONECUT1	4.26E-07	*ONECUT2;EBF2;GFRA1;INSM1;POU3F2;ISL1*
ALX1	4.26E-07	*PCDH9;ONECUT2;EBF2;GFRA1;POU3F2;ISL1*
FOXB1	4.26E-07	*ONECUT2;EBF2;GFRA1;INSM1;POU3F2;ISL1*
IRX6	4.26E-07	*PCDH9;ONECUT2;EBF2;GFRA1;INSM1;POU3F2*
NEUROG2	4.26E-07	*ONECUT2;EBF2;INSM1;MYT1;POU3F2;ISL1*
HMX1	4.26E-07	*ONECUT2;EBF2;INSM1;POU3F2;ISL1;ELAVL3*
HOXD13	4.26E-07	*ONECUT2;EBF2;GFRA1;INSM1;POU3F2;ISL1*
PAX6	4.26E-07	*ONECUT2;ELAVL4;GFRA1;INSM1;POU3F2;ISL1*
HELT	4.26E-07	*ONECUT2;EBF2;GFRA1;INSM1;POU3F2;ISL1*
FEZF1	4.26E-07	*ONECUT2;EBF2;GFRA1;INSM1;POU3F2;ISL1*
BARHL2	4.26E-07	*ONECUT2;EBF2;GFRA1;INSM1;POU3F2;ISL1*
FOXD4	4.26E-07	*ONECUT2;EBF2;GFRA1;INSM1;POU3F2;ISL1*
LBX1	4.26E-07	*ONECUT2;EBF2;GFRA1;INSM1;POU3F2;ISL1*
POU3F3	4.26E-07	*ONECUT2;EBF2;INSM1;POU3F2;ISL1;ELAVL3*
OLIG3	4.26E-07	*ONECUT2;EBF2;GFRA1;INSM1;POU3F2;ISL1*
DMRTA2	4.26E-07	*ONECUT2;ELAVL4;EBF2;INSM1;POU3F2;ISL1*
LHX8	4.26E-07	*ONECUT2;EBF2;GFRA1;INSM1;POU3F2;ISL1*
SOX11	4.26E-07	*ROBO2;ONECUT2;DCX;GFRA1;INSM1;ISL1*
DMRT3	4.26E-07	*ONECUT2;EBF2;GFRA1;INSM1;POU3F2;ISL1*
PAX7	4.26E-07	*ONECUT2;EBF2;GFRA1;INSM1;POU3F2;ISL1*
UNCX	4.26E-07	*ONECUT2;EBF2;INSM1;MYT1;POU3F2;ISL1*
LMX1A	4.26E-07	*ONECUT2;EBF2;GFRA1;INSM1;POU3F2;ISL1*
DBX1	4.26E-07	*ONECUT2;EBF2;GFRA1;INSM1;POU3F2;ISL1*
SIX3	4.26E-07	*ONECUT2;EBF2;GFRA1;INSM1;POU3F2;ISL1*
LHX4	4.26E-07	*ONECUT2;EBF2;INSM1;MDGA1;POU3F2;ISL1*
SIX6	4.26E-07	*ONECUT2;EBF2;GFRA1;INSM1;POU3F2;ISL1*
ZNF454	4.26E-07	*ROBO2;ONECUT2;ELAVL4;EBF2;GFRA1;ISL1*
NEUROD6	4.26E-07	*ROBO2;ELAVL4;DCX;INSM1;POU3F2;CHGB*
PAX1	4.26E-07	*ONECUT2;EBF2;GFRA1;INSM1;POU3F2;ISL1*
TLX3	4.26E-07	*ONECUT2;EBF2;GFRA1;INSM1;POU3F2;ISL1*
FOXB2	4.26E-07	*ONECUT2;EBF2;GFRA1;INSM1;POU3F2;ISL1*
VSX2	4.26E-07	*ONECUT2;EBF2;INSM1;MDGA1;POU3F2;ISL1*
VSX1	4.26E-07	*ONECUT2;EBF2;GFRA1;INSM1;POU3F2;ISL1*
EMX1	4.26E-07	*ONECUT2;EBF2;GFRA1;INSM1;POU3F2;ISL1*
VAX1	4.26E-07	*ONECUT2;EBF2;GFRA1;INSM1;POU3F2;ISL1*
FERD3L	4.26E-07	*ROBO2;ONECUT2;GFRA1;INSM1;POU3F2;ISL1*
LHX5	4.26E-07	*ONECUT2;EBF2;GFRA1;INSM1;POU3F2;ISL1*
POU4F3	4.26E-07	*ONECUT2;EBF2;GFRA1;INSM1;POU3F2;ISL1*
PRDM12	4.26E-07	*ONECUT2;EBF2;INSM1;MDGA1;POU3F2;ISL1*

Supplemental Table 2. Biological process enriched in the differentially expressed genes regulated by Neurog1/2 and Ascl1

Term	Adjusted P-value	Genes
neuron fate specification (GO:0048665)	1.21E-03	*POU3F2;ISL1*
cell differentiation in spinal cord (GO:0021515)	1.21E-03	*MDGA1;ISL1*
nervous system development (GO:0007399)	1.21E-03	*ROBO2;CHRNA3;DCX;GFRA1;POU3F2*
neuron fate commitment (GO:0048663)	2.31E-03	*POU3F2;ISL1*
generation of neurons (GO:0048699)	4.91E-03	*DCX;MDGA1;POU3F2*
central nervous system neuron differentiation (GO:0021953)	8.85E-03	*MDGA1;ISL1*
neuron migration (GO:0001764)	1.17E-02	*DCX;MDGA1*
central nervous system development (GO:0007417)	1.32E-02	*ROBO2;DCX;MDGA1*
axonogenesis (GO:0007409)	1.32E-02	*ROBO2;GFRA1;ISL1*
retinal ganglion cell axon guidance (GO:0031290)	4.34E-02	*ROBO2*
spinal cord motor neuron differentiation (GO:0021522)	4.34E-02	*ISL1*
norepinephrine biosynthetic process (GO:0042421)	4.34E-02	*INSM1*
norepinephrine metabolic process (GO:0042415)	4.34E-02	*INSM1*
activation of transmembrane receptor protein tyrosine kinase activity (GO:0007171)	4.34E-02	*CHRNA3*
negative regulation of chemotaxis (GO:0050922)	4.34E-02	*ROBO2*
response to ammonium ion (GO:0060359)	4.34E-02	*CHRNA3*
trigeminal nerve development (GO:0021559)	4.34E-02	*ISL1*
negative regulation of synapse organization (GO:1905809)	4.34E-02	*ROBO2*
atrial septum morphogenesis (GO:0060413)	4.34E-02	*ISL1*
sympathetic ganglion development (GO:0061549)	4.34E-02	*INSM1*
type B pancreatic cell differentiation (GO:0003309)	4.34E-02	*INSM1*
positive regulation of interleukin-1 production (GO:0032732)	4.34E-02	*ISL1*
cardiac right ventricle morphogenesis (GO:0003215)	4.34E-02	*ISL1*
negative regulation of intracellular estrogen receptor signaling pathway (GO:0033147)	4.34E-02	*ISL1*
peripheral nervous system neuron development (GO:0048935)	4.34E-02	*ISL1*
adrenal gland development (GO:0030325)	4.34E-02	*INSM1*
regulation of granulocyte macrophage CSF production (GO:0032645)	4.34E-02	*ISL1*
cranial nerve development (GO:0021545)	4.34E-02	*ISL1*
positive regulation of granulocyte macrophage CSF production (GO:0032725)	4.34E-02	*ISL1*
regulation of cell differentiation (GO:0045595)	4.34E-02	*INSM1;ISL1*
atrial septum development (GO:0003283)	4.34E-02	*ISL1*
ganglion development (GO:0061548)	4.34E-02	*INSM1*
cardiac atrium morphogenesis (GO:0003209)	4.34E-02	*ISL1*
mesonephric tubule development (GO:0072164)	4.34E-02	*ROBO2*
regulation of interleukin-1 beta production (GO:0032651)	4.34E-02	*ISL1*
pancreas development (GO:0031016)	4.34E-02	*ISL1*
regulation of neural precursor cell proliferation (GO:2000177)	4.34E-02	*INSM1*
catecholamine biosynthetic process (GO:0042423)	4.34E-02	*INSM1*
mesenchyme morphogenesis (GO:0072132)	4.34E-02	*ISL1*
cellular response to corticosteroid stimulus (GO:0071384)	4.34E-02	*ISL1*
sympathetic nervous system development (GO:0048485)	4.34E-02	*INSM1*
cellular response to glucocorticoid stimulus (GO:0071385)	4.34E-02	*ISL1*
noradrenergic neuron differentiation (GO:0003357)	4.34E-02	*INSM1*
pharyngeal system development (GO:0060037)	4.34E-02	*ISL1*
positive regulation of neural precursor cell proliferation (GO:2000179)	4.34E-02	*INSM1*
regulation of protein homodimerization activity (GO:0043496)	4.34E-02	*ISL1*
endocardial cushion morphogenesis (GO:0003203)	4.34E-02	*ISL1*
ureteric bud development (GO:0001657)	4.34E-02	*ROBO2*
acetylcholine receptor signaling pathway (GO:0095500)	4.58E-02	*CHRNA3*
endocardial cushion development (GO:0003197)	4.58E-02	*ISL1*
response to nicotine (GO:0035094)	4.87E-02	*CHRNA3*
neuromuscular synaptic transmission (GO:0007274)	4.88E-02	*CHRNA3*
outflow tract septum morphogenesis (GO:0003148)	4.88E-02	*ISL1*
regulation of smooth muscle contraction (GO:0006940)	4.88E-02	*CHRNA3*

Supplemental Table 3. crRNA, tracrRNA, and PCR primer nucleotide sequences used for this study

name	sequence (5'-3')
c3orf70a_crRNA1	cagauggauaguccaucgcaguuuuagagcuaugcuguuuug
c3orf70a_crRNA2	gcagcuugaaacauuucccgguuuuagagcuaugcuguuuug
c3orf70b_crRNA1	agauggacaguccaucacagguuuuagagcuaugcuguuuug
c3orf70b_crRNA2	gcucuggccaaaagcugugcguuuuagagcuaugcuguuuug
tracRNA	aaacagcauagcaaguuaaaauaaggcuaguccguuaucaacuugaaaaaguggcaccgagucggugcu
c3orf70a F for mutation	ggcggacagaagagtgagaa
c3orf70a R for mutation	aacccagatgacagcaccag
c3orf70b F for mutation	ctacagtggagcgcagaagg
c3orf70b R for mutation	caccagtgcagtttgaaacact
c3orf70a F for cloning and WISH	aagtggaacttgggtagccg
c3orf70a R for cloning and WISH	aggagtgtgtgcttgcactg
c3orf70b F for cloning and WISH	ggatcttaatacagatgttgtggga
c3orf70b R for cloning and WISH	agtgaagcattgtcctctacata
neurod1 F for WISH	tcgaactggaccgacaagtg
neurod1 R for WISH	ggagcgtctcgatcttggag
elavl3 F for WISH	cgacaaacggaacgaagcag
elavl3 R for WISH	catggtgacgaagccaaagc
neurod1 F for qPCR	accacgaagggcatgaaact
neurod1 R for qPCR	gtccacgtctcgttcgtctt
elavl3 F for qPCR	atcaacacgctcaacggtct
elavl3 R for qPCR	ttaccaggatgcgtgaggtg
irx3b F for qPCR	caccggctctggttctctg
irx3b R for qPCR	tgcgagctcagctggttg
actb F for qPCR	cgagcaggagatgggaacc
actb R for qPCR	caacggaaacgctcattgc

A

Neurog2 binding motif

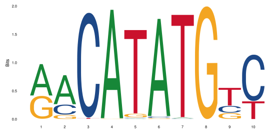

		Score
Human C3orf70	-2243 ACCATGTGGT -2252	7.0
	-1374 ACCATTTGCT -1383	6.3
Mouse C3orf70	-1926 AACATCTGTT -1935	10.4
	-1223 TGCATATGTC -1232	8.6
Zebrafish C3orf70a	- 713 AACATATGCT - 722	14.1
	-1812 GACAGATGCC -1803	10.9
Zebrafish C3orf70b	- 139 AACATATTTC - 148	10.4
	-1956 ACCACATGTC -1965	9.8

B

Ascl1 binding motif

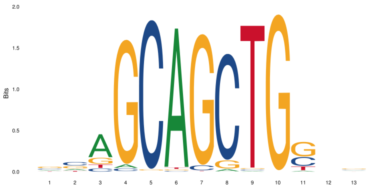

		Score
Human C3orf70	-1218 CCAGCAGGTGTGA -1230	10.9
	-1907 GGAGCAGTTGGGG -1895	9.7
Mouse C3orf70	-1398 TGGGCACCTGGGA -1386	8.8
	-1666 CAACCAGCTGTAT -1654	7.8
Zebrafish C3orf70a	- 682 GTTGCAGCTGGAA - 694	12.4
Zebrafish C3orf70b	- 59 ACTGCAGCTGTGT - 47	11.4
	- 838 GCAGCAGCAGCAA - 850	9.4

Supplemental Figure 1. Comparison of the putative binding sites for Neurog2 and Ascl1 in the promoter sequence of C3orf70. Potential binding sites for Neurog2 (A) and Ascl1 (B) in the promoters of human, mouse, and zebrafish C3orf70 are shown below the motifs.

```
c3orf70a_cDNA              ATGGCCGCTTCGGGCGGACAGAAGAGTGAGAAGTTAGATGAAGCTCAGGCTTTAGCCAGG 60
c3orf70a_NM_001126467      ATGGCCGCTTCGGGCGGACAGAAGAGTGAGAAGTTAGATGAAGCTCAGGCTTTAGCCAGG 60
                           ************************************************************

c3orf70a_cDNA              AGTTGCGCAGGACGACCGGACTTTCTGCCCTGCGATGGACTATCCATCTGCGCCACACAC 120
c3orf70a_NM_001126467      AGTTGCGCAGGACGACCGGACTTTCTGCCCTGCGATGGACTATCCATCTGCGCCACACAC 120
                           ************************************************************

c3orf70a_cDNA              AGCCACGGGAAATGTTTCAAGCTGCACTGGTGCTGTCATCTGGGTTGGTGCCACTGTAAA 180
c3orf70a_NM_001126467      AGCCACGGGAAATGTTTCAAGCTGCACTGGTGCTGTCATCTGGGTTGGTGCCACTGTAAA 180
                           ************************************************************

c3orf70a_cDNA              TACGTCTACCAACCTATGACCAGCGTATGCCAACTTCCCAGCACAGCGGTTCCAGTCGCT 240
c3orf70a_NM_001126467      TACGTCTACCAACCTATGACCAGCGTATGCCAACTTCCCAGCACAGCGGTTCCAGTCGCT 240
                           ************************************************************

c3orf70a_cDNA              CCCTCAGGTCATACGCACACTATGAACTTGTCCATCTCGCTGGCTGAACGTTTTCTCAGG 300
c3orf70a_NM_001126467      CCCTCAGGTCATACGCACACTATGAACTTGTCCATCTCGCTGGCTGAACGTTTTCTCAGG 300
                           ************************************************************

c3orf70a_cDNA              ACAGCTCCCAAGTTCCAGGCTCCACCCTGCCCTGAGTCTCCCAAGTTCTGTGTCATCTCT 360
c3orf70a_NM_001126467      ACAGCTCCCAAGTTCCAGGCTCCACCCTGCCCTGAGTCTCCCAAGTTCTGTGTCATCTCT 360
                           ************************************************************

c3orf70a_cDNA              GACCTCTTTGTGGATGATTACATGGTCAAGCGTATCAATGGTAAAATGTGTTACGTGCAA 420
c3orf70a_NM_001126467      GACCTCTTTGTGGATGATTACATGGTCAAGCGTATTAATGGTAAAATGTGTTACGTGCAA 420
                           *********************************** ************************

c3orf70a_cDNA              AGGCCTCCCCCGCCTACACCAAATCCCACACACCAACCTCAAACGGCGGCACCTCAGCCT 480
c3orf70a_NM_001126467      AGGCCTCCCCCGCCTACACCAAATCCCACACACCAACCTCAAACGGCGGCACCTCAGCCT 480
                           ************************************************************

c3orf70a_cDNA              GTGCCCCAACGTTCCAAAAACAGTCATCAAGTGGGCCCAACTGTTAAACAAGGCCAAGCA 540
c3orf70a_NM_001126467      GTGCCCCAACGTTCCAAAAACAGTCATCAAGTGGGCCCAACTGTTAAACAAGGCCAAGCA 540
                           ************************************************************

c3orf70a_cDNA              AAGGAGAAGATCTCAGCCCCTAAAATGGACCACTGCTCCTCTCCCTCCAGCTCAGAGGAC 600
c3orf70a_NM_001126467      AAGGAGAAGATCTCAGCCCCTAAAATGGACCACTGCTCCTCTCCCTCCAGCTCAGAGGAC 600
                           ************************************************************

c3orf70a_cDNA              TCTGGAATCAATGCATTGGGTTTGCACTACATGGAATCATGCGACGAGGACTCTTGTGTA 660
c3orf70a_NM_001126467      TCTGGAATCAATGCATTGGGTTTGCACTACATGGAATCATGCGACGAGGACTCTTGTGTA 660
                           ************************************************************

c3orf70a_cDNA              GATGACGATGACGAGGAAGAAGAGGACGATGAGCTGAGTACTGATGGAAACTCCAGCCCA 720
c3orf70a_NM_001126467      GATGACGATGACGAGGAAGAAGAGGACGATGAGCTGAGTACTGATGGAAACTCCAGCCCA 720
                           ************************************************************

c3orf70a_cDNA              GGTAGCTTCTGGGACCAGGACGAGTGCACTTTACTATCACCATCTAAATCAATCGTTGAG 780
c3orf70a_NM_001126467      GGTAGCTTCTGGGACCAGGACGAGTGCACTTTACTATCACCATCTAAATCAATCGTTGAG 780
                           ************************************************************

c3orf70a_cDNA              ATCATTGAGAAGATTGAGACTACAGTTTGA 810
c3orf70a_NM_001126467      ATCATTGAGAAGATTGAGACTACAGTTTGA 810
                           ******************************
```

Supplemental Figure 2. Comparison of the nucleotide sequences of c3orf70a open reading frame determined in this study and human sequence NM_001126467 in the NCBI Reference Sequence Database. Red arrowhead indicates the single nucleotide difference between the cDNA and reference sequences.

```
c3orf70b_cDNA             ATGGCCTACAGTGGAGCGCAGAAGGCTTTGAAGAGTGATAAGTTAGATGAAGCTCAAGCT 60
c3orf70b_NM_001089454     ATGGCCTACAGTGGAGCGCAGAAGGCTTTGAAGAGTGATAAGTTAGATGAAGCTCAAGCT 60
                          ************************************************************

c3orf70b_cDNA             CTGGCCAAAAGCTGTGCGGGCAGACCGGACTTCCTCCCCTGTGATGGACTGTCCATCTGC 120
c3orf70b_NM_001089454     CTGGCCAAAAGCTGTGCGGGCAGACCGGACTTCCTCCCCTGTGATGGACTGTCCATCTGC 120
                          ************************************************************

c3orf70b_cDNA             GCAACACACAGCCATGGAAAGTGTTTCAAACTGCACTGGTGCTGCCATTTGGGCTGGTGT 180
c3orf70b_NM_001089454     GCAACACACAGCCATGGAAAGTGTTTCAAACTGCACTGGTGCTGCCATTTGGGCTGGTGT 180
                          ************************************************************

c3orf70b_cDNA             CACTGTAAATATGTGTACCAGCCCATGACCAACGTGGCTCAGCTGCCCAGCACACCTGTG 240
c3orf70b_NM_001089454     CACTGTAAATATGTGTACCAGCCCATGACCAACGTGGCTCAGCTGCCCAGCACACCTGTG 240
                          ************************************************************

c3orf70b_cDNA             CCTGCGGCTCCGTCTGACTGCCCCGACACCATTGATCTGTCCATTTCTCTGACCGAACGC 300
c3orf70b_NM_001089454     CCTGCGGCTCCGTCTGACTGCCCCGACACCATTGATCTGTCCATTTCTCTGACCGAACGC 300
                          ************************************************************

c3orf70b_cDNA             TTCCTCCGCATCTCCCCCTGCTTCCAGCCTCCACCCTGTCCAGAATCGCCCAAATACTGC 360
c3orf70b_NM_001089454     TTCCTCCGCATCTCCCCCTGCTTCCAGCCTCCACCCTGTCCAGAATCGCCCAAATACTGC 360
                          ************************************************************

c3orf70b_cDNA             AACATCGCCGAACTCTTTATTGACGACTATATCGTTAAACGAATCAACGGCAAGATGTGC 420
c3orf70b_NM_001089454     AACATCGCCGAACTCTTTATTGACGACTATATCGTTAAACGAATCAACGGCAAGATGTGC 420
                          ************************************************************

c3orf70b_cDNA             TACGTGCAGCGGCCCCAGGCTCATGTGGAGCCTGCTCAAATGAACCCAATTCAGAAACAG 480
c3orf70b_NM_001089454     TACGTGCAGCGGCCCCAGGCTCATGTGGAGCCTGCTCAAATGAACCCAATTCAGAAACAG 480
                          ************************************************************

c3orf70b_cDNA             CACACCGAAGACAAACAGATTGTTGAGGAGACTGTCAAAGGACCAAAAATGGGCCACTGC 540
c3orf70b_NM_001089454     CACACCGAAGACAAACAGATTGTTGAGGAGACTGTCAAAGGACCAAAAATGGGCCACTGC 540
                          ************************************************************

c3orf70b_cDNA             TCTTCGCCCTCTACCTCCGAGGACTCAGGGATCAACGCTTTGGGAGGACACTTCCTGGAG 600
c3orf70b_NM_001089454     TCTTCGCCCTCTACCTCCGAGGACTCAGGGATCAACGCTTTGGGAGGACACTTCCTGGAG 600
                          ************************************************************

c3orf70b_cDNA             TCCTGCGAGGAAGAGTCTGAGGAAGAGGATGAGCTCAGCACGGATGGACACTCCAGTCCG 660
c3orf70b_NM_001089454     TCCTGCGAGGAAGAGTCTGAGGAAGAGGATGAGCTCAGCACGGATGGACACTCCAGTCCA 660
                          *********************************************************** 

c3orf70b_cDNA             GGGAGTCTATGGGACCAGGACGAATGCACCCTGCTTTCCCCTTCCAAATCCATGGTGGAG 720
c3orf70b_NM_001089454     GGGAGTCTATGGGACCAGGACGAATGCACCCTGCTTTCCCCTTCCAAATCCATGGTGGAG 720
                          ************************************************************

c3orf70b_cDNA             ATTATTGAAAACATCGAAACGACCGTGTGA 750
c3orf70b_NM_001089454     ATTATTGAAAACATCGAAACGACCGTGTGA 750
                          ******************************
```

Supplemental Figure 3. Comparison of the nucleotide sequences of c3orf70b open reading frame determined in this study and NM_001089454 in the NCBI Reference Sequence Database. Red arrowhead indicates the single nucleotide difference between the cDNA and reference sequences.

```
c3orf70a_cDNA            MAASGGQKSEKLDEAQALARSCAGRPDFLPCDGLSICATHSHGKCFKLHWCCHLGWCHCK 60
c3orf70a_NM001126467     MAASGGQKSEKLDEAQALARSCAGRPDFLPCDGLSICATHSHGKCFKLHWCCHLGWCHCK 60
                         ************************************************************

c3orf70a_cDNA            YVYQPMTSVCQLPSTAVPVAPSGHTHTMNLSISLAERFLRTAPKFQAPPCPESPKFCVIS 120
c3orf70a_NM001126467     YVYQPMTSVCQLPSTAVPVAPSGHTHTMNLSISLAERFLRTAPKFQAPPCPESPKFCVIS 120
                         ************************************************************

c3orf70a_cDNA            DLFVDDYMVKRINGKMCYVQRPPPPTPNPTHQPQTAAPQPVPQRSKNSHQVGPTVKQGQA 180
c3orf70a_NM001126467     DLFVDDYMVKRINGKMCYVQRPPPPTPNPTHQPQTAAPQPVPQRSKNSHQVGPTVKQGQA 180
                         ************************************************************

c3orf70a_cDNA            KEKISAPKMDHCSSPSSSEDSGINALGLHYMESCDEDSCVDDDDEEEEDDELSTDGNSSP 240
c3orf70a_NM001126467     KEKISAPKMDHCSSPSSSEDSGINALGLHYMESCDEDSCVDDDDEEEEDDELSTDGNSSP 240
                         ************************************************************

c3orf70a_cDNA            GSFWDQDECTLLSPSKSIVEIIEKIETTV 269
c3orf70a_NM001126467     GSFWDQDECTLLSPSKSIVEIIEKIETTV 269
                         *****************************
```

```
c3orf70b_cDNA            MAYSGAQKALKSDKLDEAQALAKSCAGRPDFLPCDGLSICATHSHGKCFKLHWCCHLGWC 60
c3orf70b_NM_001089454    MAYSGAQKALKSDKLDEAQALAKSCAGRPDFLPCDGLSICATHSHGKCFKLHWCCHLGWC 60
                         ************************************************************

c3orf70b_cDNA            HCKYVYQPMTNVAQLPSTPVPAAPSDCPDTIDLSISLTERFLRISPCFQPPPCPESPKYC 120
c3orf70b_NM_001089454    HCKYVYQPMTNVAQLPSTPVPAAPSDCPDTIDLSISLTERFLRISPCFQPPPCPESPKYC 120
                         ************************************************************

c3orf70b_cDNA            NIAELFIDDYIVKRINGKMCYVQRPQAHVEPAQMNPIQKQHTEDKQIVEETVKGPKMGHC 180
c3orf70b_NM_001089454    NIAELFIDDYIVKRINGKMCYVQRPQAHVEPAQMNPIQKQHTEDKQIVEETVKGPKMGHC 180
                         ************************************************************

c3orf70b_cDNA            SSPSTSEDSGINALGGHFLESCEEESEEEDELSTDGHSSPGSLWDQDECTLLSPSKSMVE 240
c3orf70b_NM_001089454    SSPSTSEDSGINALGGHFLESCEEESEEEDELSTDGHSSPGSLWDQDECTLLSPSKSMVE 240
                         ************************************************************

c3orf70b_cDNA            IIENIETTV 249
c3orf70b_NM_001089454    IIENIETTV 249
                         *********
```

Supplemental Figure 4. Comparison of the inferred amino acid sequences of c3orf70a and c3orf70b open reading frames obtained in this study and NCBI reference sequences NM_001126467 and NM_001089454.
